# Identification of cuproptosis-related molecular classification and characteristic genes in ulcerative colitis

**DOI:** 10.1016/j.heliyon.2024.e24875

**Published:** 2024-01-19

**Authors:** Xinyu Bai, Fengrui Zhang, Chan Zhou, Jingxian Yan, Hao Liang, Rui Zhu, Min Gong, Huixian Song, Junkun Niu, Yinglei Miao

**Affiliations:** a Kunming Medical University, Kunming, China; b Department of Gastroenterology, The First Affiliated Hospital of Kunming Medical University, Kunming, China; c Yunnan Province Clinical Research Center for Digestive Diseases, Kunming, China

**Keywords:** Ulcerative colitis, Cuproptosis, Molecular clusters, Machine learning, Immune infiltration

## Abstract

Ulcerative colitis (UC) is a refractory inflammatory disease with imbalances in intestinal mucosal homeostasis. Cuproptosis serves as newly identified programmed cell death (PCD) form involved in UC. In the study, UC-related datasets were extracted from the Gene Expression Omnibus (GEO) database. A comparison of UC patients and healthy controls identified 11 differentially expressed cuproptosis-related genes (DE-CRGs), where FDX1, LIAS, and DLAT were differentially expressed in UC groups from the mouse models and clinical samples, with their expression correlating with disease severity. By comprehending weighted gene co-expression network analysis (WGCNA) and differential expression analysis, the key genes common to the module genes relevant to different cuproptosis-related clusters and differentially expressed genes (DEGs) both in different clusters and patients with and without UC were identified using several bioinformatic analysis. Furthermore, the mRNA levels of four characteristic genes with diagnostic potential demonstrated significant decrease in both mouse models and clinical UC samples. Our discoveries offer a theoretical foundation for cuproptosis effect in UC.

## 1 Introduction

Ulcerative colitis (UC), a persistent and non-specific inflammatory disease, affects intestines, primarily marked by diarrhea, bloody stools, abdominal pain. Multiple factors are thought to be participated in UC pathogenesis, including genetic susceptibility, immune dysregulation, and environmental factors [1]. Imbalances in intestinal mucosal homeostasis were reported to work critically within UC occurrence and progression [2]. Excessive apoptotic and necroptotic death of intestinal epithelial cells (IECs) have been detected in the intestinal mucosa tissue of UC patients, resulting in intestinal epithelial barrier disruption, as well as intestinal mucosal homeostasis breakdown [[3], [4], [5]]. Autophagy of intestinal immune cells may also regulate intestinal immune homeostasis by affecting both innate and adaptive immunity [6]. The two classical forms of programmed cell death (PCD), autophagy and apoptosis, can influence intestinal homeostasis and play roles in the development of disease. Although several novel cell death pathways, inclusive of ferroptosis and cuproptosis, were identified in recent years, their functional roles in UC remain unclear. Exploring the relationships between UC and these novel cell death pathways may increase our comprehension of cell death in UC, and unveil novel therapeutic targets.

Copper has been reported that it may be abnormally metabolized in patients with UC, where serum concentrations of copper ions (Cu^2+^) in UC cohort surpassed healthy controls [7,8]. High concentrations of copper ions-induced toxicity targeting regulatory cell death, inclusive of macroautophagy/autophagy, ferroptosis, apoptosis, pyroptosis, cuproptosis, and paraptosis [9]. Although all were copper ion-induced regulatory cell death, cuproptosis, which is caused by excess copper directly binds to tricarboxylic acid cycle's lipoylated components and results in cellular stress and ultimately cell death [10], fundamentally apart from autophagy, pyroptosis, and apoptosis [11]. Key regulators in cuproptosis, inclusive of DLAT, FDX1, and LIAS, were found to be notably altered in UC animal models, and cuproptosis was closely correlated with UC immune cell infiltration [12,13], providing a rationale for cuproptosis in the colonic tissue of these patients. Further, bioinformatic analyses targeting molecular subtyping of UC were conducted to research association between cuproptosis-related genes (CRGs) and UC progression. For example, it has been identified two key cuproptosis-related clusters and detected five UC-cluster-related biomarkers by weighted gene co-expression network analysis (WGCNA) and four machine models [14]. Yang et al. examined the differential expression of three CRGs between UC and controls via the public datasets and a mouse model of UC [15]. Tang et al. constructed a seven-DE-CRGs-based nomogram for predicting the risk of UC [16]. In all, the findings indicate, cuproptosis plays a determinant in UC.

**Fig. 1:**
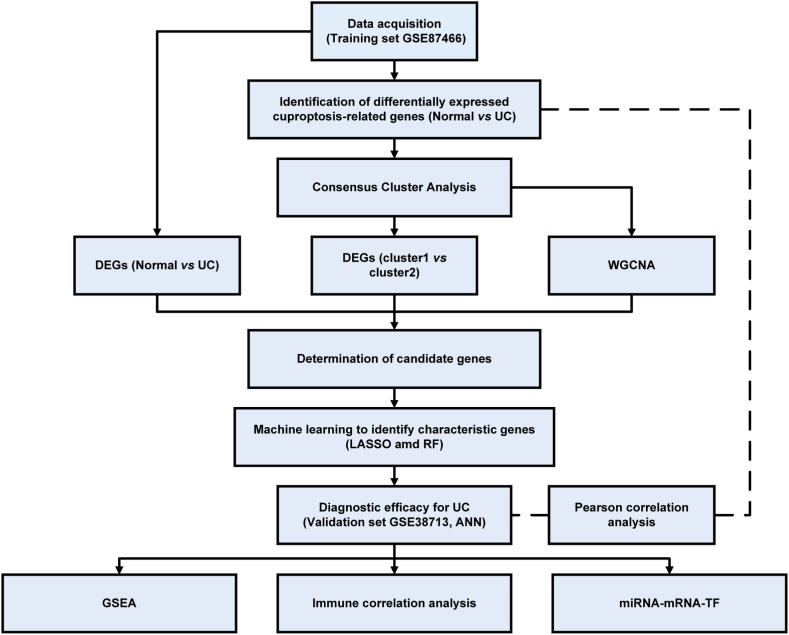
Flowchart of the present study.

Inspired by these studies, the present study targeted DE-CRGs in UC samples and healthy controls based on information from public databases, and measured cuproptosis-related regulators protein expression in the dextran sodium sulfate (DSS)-induced colitis mouse model, and clinical UC patients with different disease severity. Consensus clustering analysis based on DE-CRGs expression profiles was used to classify patients with UC from training set into distinct molecular phenotypes. Further, key characteristic genes of UC were screened basing on differential expression analysis and WGCNA, and the mRNA levels of which were examined in the mouse and clinical cohorts. Moreover, comparisons of the biological functions and immune cell infiltration in different clusters were completed. These discoveries offer a glimpse into the potential cuproptosis participation in UC progression. The study's methodology is demonstrated in Fig. 1.

## 2 Materials and methods

### 2.1 Data acquisition

Three indicators were considered in the screening of the UC datasets: the date of publication; the size of the sample (with a preference for datasets with larger sample sizes to be used as the training set); and the gene count (with a preference for datasets with a larger number of genes). Two independent datasets (GSE87466 and GSE38713) were extracted from GEO database (https://www.ncbi.nlm.nih.gov/geo/). GSE87466 dataset, consisting of 21 healthy controls and 87 UC patients, being applied as training set, and the GSE38713 dataset, consisting of 13 healthy individuals and 30 UC subjects, was used as validation set. The 19 individually assayed CRGs (PDHB, MTF1, DBT, SLC31A1, GLS, CDKN2A, ATP7A, DLD, ATP7B, DLST, NLRP3, PDHA1, GCSH, FDX1, LIAS, LIPT1, DLAT, LIPT2, and NFE2L2) had been previously identified [17].

### 2.2 Detection and DE-CRGs functional enrichment analysis in UC and control cohorts

The mRNA expressions encoded by the 19 CRGs described above was compared in UC and control within training set using Wilcox.test (Adjustment on *P* value using Benjamini-Hochberg method) and drawn by the “ggplot 2” R package, and the correlations between DE-CRG expressions were assessed by “corrplot” R package (version 0.89). DE-CRGs biological functions were searched by Gene Ontology (GO), Kyoto Encyclopedia of Genes and Genomes (KEGG) pathway analyses by “clusterProfiler” R package [18].

### 2.3 Consensus clustering of UC cohorts and functional enrichment analysis of different clusters

Using DE-CRGs identified, consensus cluster analysis was completed utilizing “ConsensusClusterPlus” R package (version 1.54.0) to identify different sample clusters [19], and principal component analysis (PCA) analysis within clusters was completed employing “PCA” R package. DE-CRGs differential expression within various clusters were compared by Wilcox.test, and Benjamini-Hochberg method got applied to perform a multiple-testing correction for the *P*-values. Further, the potential pathways that included characteristic genes in UC were evaluated by GSEA (version 3.42.2). “c2.cp.kegg.v7.4.symbols.gmt” and “c5.go.bp.v7.4.symbols.gmt” were taken as reference to explore the differential gene sets of all genes in the training set between cluster 1 and cluster 2. Remaining parameters are set as follows: “Metric for ranking genes” = log 2 ratio of class, “Gene list sorting mode” = real, “Gene list ordering mode” = descending. The items with |NES| > 1 and NOM *p*-val <0.05 were taken statistically significant.

### 2.4 Human colonic tissue samples

Fifteen active UC patients and five healthy individuals were enrolled at the Gastroenterology Department of the First Affiliated Hospital of Kunming Medical University. UC severity was classified as mild, moderate, or severe based on Mayo Scores. For each severity level, five patients were selected. All patients had left-sided colitis (E2), and had received only 5-aminosalicylic acid (5-ASA) within the three months prior to sample collection. Patients requiring additional therapeutic agents underwent biopsies as soon as possible before medication administration, in order to minimize potential confounding effects of other medications on the study outcomes. All colonoscopies were conducted by skilled endoscopists with specialized training in Inflammatory Bowel Disease (IBD). Colonic biopsies were procured from the sigmoid colon, with each biopsy containing six pieces. Every participant enrolled within the research handed in written informed consent. The utilization of biopsy samples received approval from Ethics Committee of The First Affiliated Hospital of Kunming Medical University.

### 2.5 Animal experiments

Twenty male C57BL/6J mice, aged 6–8 weeks and weighing 18–22 g, were procured from the Experimental Animal Center of Kunming Medical University. Following a meticulous one-week acclimatization period, these murine subjects underwent randomization into two distinct groups, each comprising ten individuals. In the experimental paradigm, the cohort designated as the DSS cohort was subjected to 3 % DSS solution (MP Biomedicals, United States) administration integrated into their drinking water for 7 days. Concurrently, counterparts in the control cohort received a regimen of distilled water [20]. Study culmination involved the sacrifice of all mice on the 8th day. Critical to ethical considerations, the protocol governing these animal experiments received the explicit approval of the Animal Ethics Committee of Kunming Medical University (KMMU2021187).

### 2.6 Western blotting

Clinical samples and mice tissue were added to RIPA lysis buffer with 1 % protease inhibitors, and homogenized using an electric homogenizer. Western blotting experiments were completed in aforementioned way [21]. Antibodies used in the present study included anti-LIAS (1:3000, Proteintech), anti-DLAT (1:5000, Proteintech), anti-FDX1 (1:500, Lifespan), and anti-GAPDH (1:5000, Proteintech). These results were evaluated using Image J software.

### 2.7 Selection of hub genes correlated with different clusters and normal group

WGCNA was completed for identification of genes related to different UC clusters and normal individuals. First, clustering analysis of all samples in the training set showed that there were no outliers (Fig. S1A). For the construction of co-expression network approaching scale-free distribution, the optimal soft threshold (β) was determined as 28 (Fig. S1B). The adjacency and similarity between genes are calculated, and the dynamic tree cutting algorithm confirmed a total of nine modules using the degree of dissimilarity when minModuleSize was 70 and MEDissThres was 0.25 (Fig. S1C). Further, correlations between modules and traits (normal group and different clusters) were computed, where modules with |cor| > 0.3 and *P* < 0.05 were selected. The module genes with |gene significance (GS)| > 0.2, |model membership (MM)| > 0.8, and *P* < 0.05 were further identified as hub genes for subsequent analyses.

### 2.8 Identification and construction of protein-protein interactions (PPI) networks of candidate genes

Differential expression analysis was performed on different clusters and cohorts between UC and normal groups in training set by “limma” R package (version 3.42.2), respectively [22]. The screening conditions for DEGs were |log_2_ (fold change)| > 0.585 and *P* < 0.05 (where Benjamini-Hochberg method was conducted for correction for multiple testing) [23]. The DEGs in the UC and normal groups were noted as DEG1, and the DEGs of the different clusters were noted as DEG2. Ultimately, DEG1, DEG2, and the hub genes were taken as intersections by Veen tool to obtain intersecting genes, noted as candidate genes for further analysis. The Search Tool for the Retrieval of Interacting Genes (STRING) (http://string-db.org/) was employed to construct candidate genes PPI network, with downloaded analytical results possessing a confidence score greater than 0.4. Following this, the visualization of the PPI network was accomplished by Cytoscape software.

### 2.9 Selection and functional enrichment analysis of characteristic genes

From candidate genes, characteristic genes were selected by two machine learning algorithms, namely least absolute shrinkage and selection operator (LASSO) and random forest (RF). Diagnostic efficacy was predicted by receiver operating characteristic (ROC) curves utilizing the “pROC” R package and decision curve analysis (DCA) curves by “rmda” R package [24]. And meanwhile, logistic regression (LR) and artificial neural network (ANN) models were built for validation of the characteristic genes. Next, potential pathways that included characteristic genes in UC were evaluated by single gene GSEA via the “clusterProfiler” R package. Following the Pearson correlation coefficient was calculated between each characteristic gene and 11 DE-CRGs in the training set, the corresponding list of ranked genes were obtained according to the correlation coefficients from large to small. Likewise, GO and KEGG pathway enrichment analysis were performed using GSEA analysis. Further, multiple testing correction was completed by Benjamini-Hochberg methodology for a corrected *P-*value (*p*. adjust), where *p*. adjust <0.05 was considered significant.

### 2.10 Immune cell infiltration scoring

CIBERSORT algorithm was utilized to assess 22 types of immune cells phenotypes proportions in each patient from different clusters in the training set combined with the LM22 gene signature matrix (*P* < 0.05) [25,26]. The differences of immune cells infiltrations between clusters were calculated using the Wilcox.test and violin plots were generated using the “vioplot” R package. Characteristic genes correction with differentially expressed immune cell populations were analyzed by the Spearman algorithm.

### 2.11 Construction of a miRNA-mRNA-transcription factors (TFs) network of characteristic genes

For further comprehension of characteristic genes mechanisms in UC, we constructed a miRNA-mRNA-TF network. Firstly, we used mirwalk database 3.0 and miRTarBase to predict miRNAs that bind to characteristic genes. Further, the miRNAs obtained via the two ways were taken to intersect to obtain the miRNAs of characteristic genes. The TFs of characteristic genes were predicted through hTFtarget database. Finally, the miRNA-mRNA-TF network was constructed sequentially using Cytoscape software [27].

### 2.12 Quantitative reverse Transcription polymerase chain reaction (qRT-PCR)

Platinum® SYBR® Green qPCR kit (TaKaRa) and the LightCycler 480 System (Roche) were used for qRT-PCR reactions following professional instructions. Primer sequences were designed by Takara Bio, Inc. (Table 1).

**Table 1:** The primer sequences were used in this study.

Genes	Organisms	Forward primer	Reverse primer
STRADB	Mus musculus	TTGGAGCAGTACAAGGGTTGAACTA	GTCACCAGGCCATCACCAGA
AHCYL2	Mus musculus	TGCCCTAATGCTGGAACCAA	TCCTTGTCTCCAGGTAGCTAGAGTG
SRI	Mus musculus	CCTTGCTATGTATGTGCCTTGGA	TTAACCATTGTTAGCTGCCAAACTG
GLTP	Mus musculus	CATCAAGGCAGACATAAGCGGTA	TTCTGCAGGGTCTTGAACTTGG
β-actin	Mus musculus	CACCATTGGCAATGAGCGGTTC	AGGTCTTTGCGGATGTCCACGT
STRADB	Homo sapiens	TCTCCAGTGGAACTCACACAGTAA	GGGACAATAAACTGCTTGCTGATG
AHCYL2	Homo sapiens	ACCCAAGCCTGCACTATGGA	GCTTCTGGTCAGCAAACTGGA
SRI	Homo sapiens	GGACAAACTCAGGATCCGCTGTA	GCCGGCAAGTCTCCAGGTTA
GLTP	Homo sapiens	AGTGTTTACTCCCATCAAGGCAGAC	TTGGGCCACTCTGCTCCATA
β-actin	Homo sapiens	TGGCACCCAGCACAATGAA	CTAAGTCATAGTCCGCCTAGAAGCA

### 2.13 Statistical analysis

Categorical data were presented as numerical values (percentage), while continuous data were delineated as mean ± SD. Statistical comparisons were executed through ANOVA and LSD-T tests. In-depth statistical analyses of bioinformatics data were completed by R software (version 4.1.2), while experimental data underwent scrutiny using SPSS 25.0 statistical software. Significance was established at a *p* value < 0.05. In the graphical representations, significance levels were denoted as follows: *p < 0.05, **p < 0.01, ***p < 0.001, and ****p < 0.0001.

## 3 Results

### 3.1 Selection and functional enrichment of DE-CRGs

**Fig. 2:**
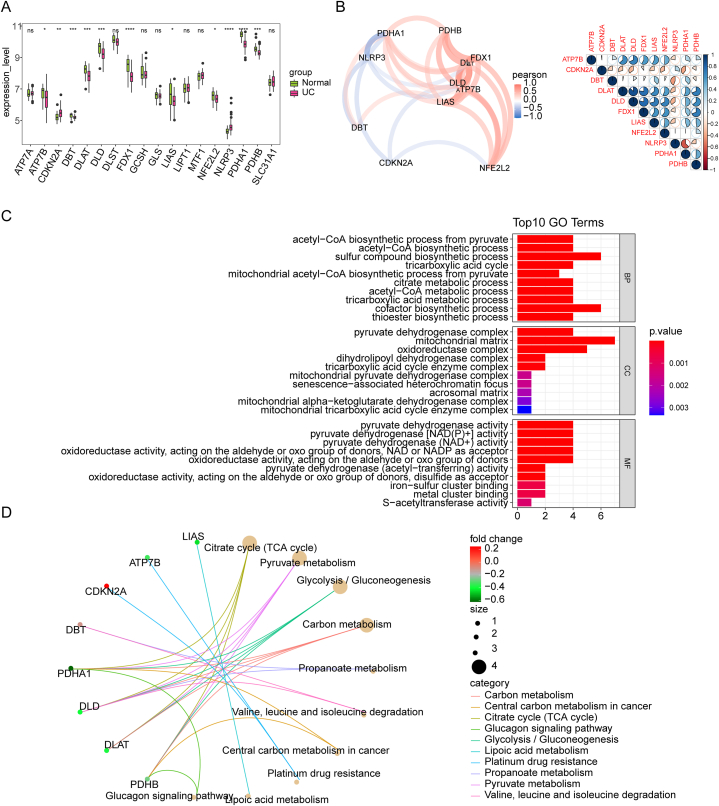
Identification and functional analysis of DE-CRGs in UC. (A) Boxplots illustrating the expression profiles of DE-CRGs in UC patients compared to controls. (B) Pairwise correlation analysis elucidating the interplay among the expression patterns of the 11 DE-CRGs. (C) Top 10 enriched biological elements disclosed through GO analysis. (D) KEGG analysis enriched items.

To identify DE-CRGs in UC, 19 CRGs mRNA levels were compared in UC and healthy individuals in the training set, and eleven DE-CRGs were detected, with ATP7B, DBT, DLAT, DLD, FDX1, LIAS, NFE2L2, PDHA1, and PDHB being significantly decreased, and the levels of CDKN2A and NLRP3 being escalated in UC patients in comparison to controls (Fig. 2A). Evaluation of the pairwise correlation of DE-CRGs levels showed a significant association between DLD and DLAT expression (Fig. 2B). Besides, evaluation of GO items showed that these DE-CRGs were considerably enriched in the biosynthesis of acetyl-CoA from pyruvate (BP), the pyruvate dehydrogenase complex (CC), and pyruvate dehydrogenase activity (MF) (Fig. 2C). KEGG analysis discovered, these DE-CRGs were enriched in pyruvate metabolism, glycolysis/gluconeogenesis, TCA cycle (Fig. 2D).

### 3.2 Expression of DE-CRGs in DSS-induced colitis and UC patients

**Fig. 3:**
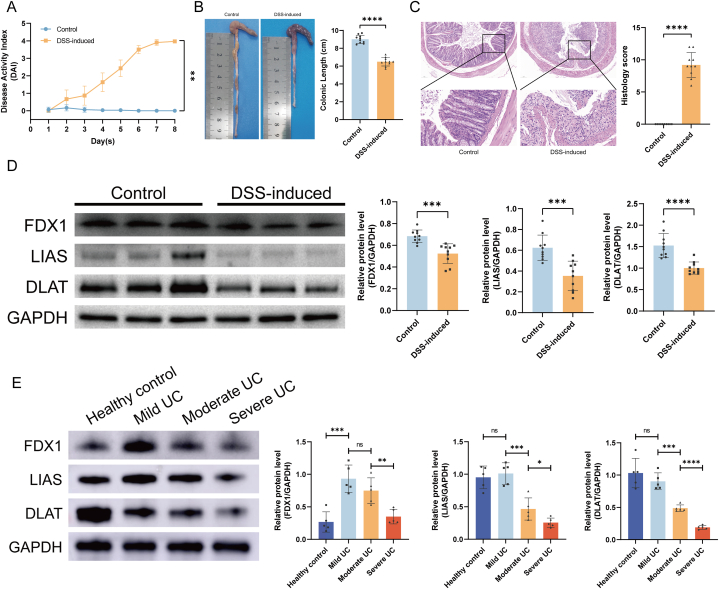
Expression of DE-CRGs in the mouse models and clinical samples. (A) Disease activity index (DAI) scores. (B) Representative pictures of the colon and statistics of colon lengths. (C) Images of HE-stained colon sections and histology scores of the two groups. (D) Western blotting analysis of FDX1, LIAS, and DLAT proteins in DSS-induced and control mice. The original Western blotting images were available in the supplementary file. (E) Western blotting analysis of FDX1, LIAS, and DLAT proteins in clinical samples from UC patients and healthy controls. The original Western blotting images were available in the supplementary file.

An animal model of UC was established by administering 3 % DSS to mice, with the DAI score being significantly increased (Fig. 3A) and colon length shorter (Fig. 3B) in DSS-induced than in control mice. Histopathological analysis of colonic tissue sections from the control group showed that the intestinal structure was intact and that IECs were arranged neatly (Fig. 3C). In contrast, sections from the DSS-induced group showed structural disorder of the colon, with obvious loss of IECs, and infiltration of large numbers of inflammatory cells (Fig. 3C).

A comparative analysis of the protein expression of cuproptosis-associated regulators, namely FDX1, LIAS, and DLAT, between the two experimental groups revealed a notable drop in the levels of these proteins in DSS-induced mice compared to the control counterparts. This observation aligns with the findings from the public dataset, as illustrated in Fig. 3D. In an endeavor to ascertain key cuproptosis regulators and disease severity correlation, FDX1, LIAS, and DLAT protein expressions was scrutinized in UC patients exhibiting varying degrees of disease severity. In comparison to healthy controls, the protein levels of LIAS and DLAT exhibited a declining trend with increasing disease severity in UC patients, excluding mild UC cases. Similarly, the expression trend of FDX1 demonstrated a decrease from moderate UC to severe UC. Intriguingly, FDX1 expression in patients with mild UC surpassed control group, adding a nuanced layer to the complexity of these regulatory dynamics, as depicted in Fig. 3E.

### 3.3 Classification of UC clusters based on eleven DE-CRGs

**Fig. 4:**
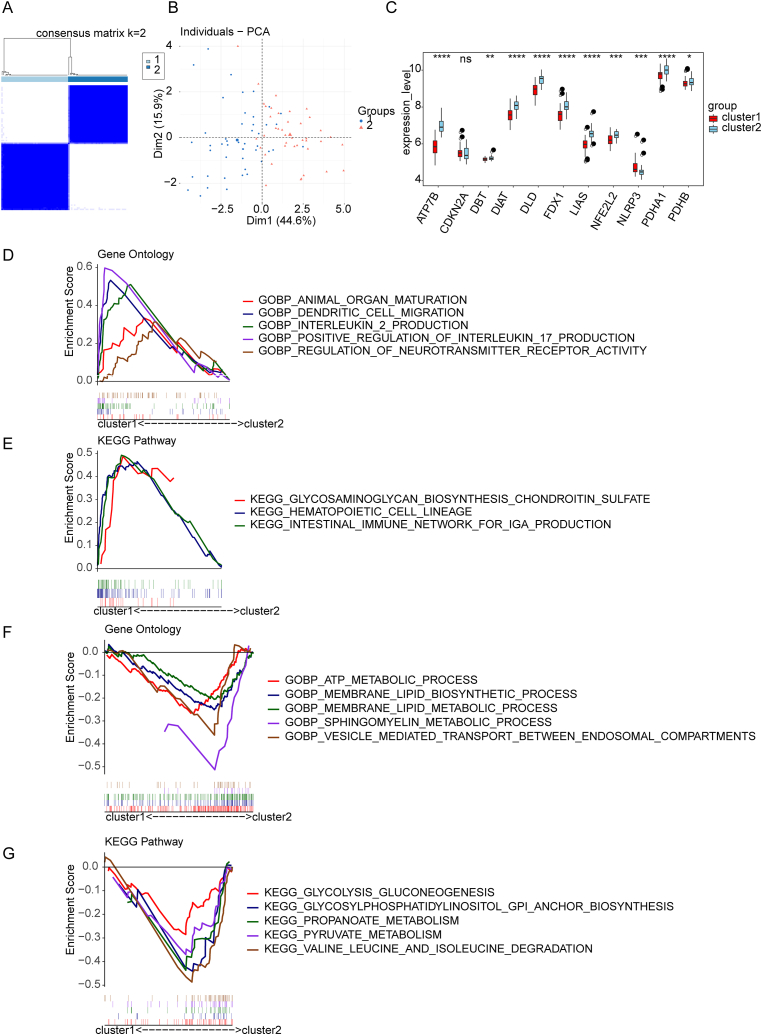
Classification of UC subtypes and functional analysis of differential cuproptosis clusters. (A) Consensus matrix (k = 2) classification of two clusters. (B) PCA analysis of clusters 1 and 2. (C) Boxplots showing eleven DE-CRGs expressions in two clusters. (D, F) GSEA enrichment analysis of GO BP items in clusters 1 and 2. (E, G) GSEA enrichment analysis of KEGG pathways in clusters 1 and 2.

Next, according to expression patterns of eleven DE-CRGs, consensus clustering analysis revealed that the clustering effect was most stable when the clustering number K = 2, hence the UC samples in the training set were classified into two different clusters (cluster 1 = 46, cluster 2 = 41) (Fig. 4A, Table S1). PCA analysis showed that the two clusters differed significantly in distribution patterns (Fig. 4B), as did in the expression of eleven DE-CRGs (Fig. 4C). Specifically, the levels of ATP7B, DBT, DLAT, DLD, FDX1, LIAS, NFE2L2, PDHA1, and PDHB were increased in cluster 2, whereas the level of NLRP3 was escalated in cluster 1. Subsequently, GSEA analysis was completed to determine the disparities in biological pathways between two clusters. In GO BP items, cluster 1 was enriched in positive regulation of interleukin 17 (IL-17) production, dendritic cell migration, and interleukin 2 (IL-2) production (Fig. 4D), whereas cluster 2 was enriched in processes of ATP metabolism, membrane lipid biosynthesis, and membrane lipid metabolism (Fig. 4F). KEGG pathway analysis suggested, cluster 1 enriched within glycosaminoglycan biosynthesis and chondroitin sulfate pathways, as well as in pathways associated with hematopoietic cell lineages and intestinal immune network involved in IgA production (Fig. 4E). In contrast, and cluster 2 was enriched in glycosylphosphatidylinositol (GPI) anchor biosynthesis, propanoate metabolism, and pyruvate metabolism (Fig. 4G).

### 3.4 Determination of key candidate genes

**Fig. 5:**
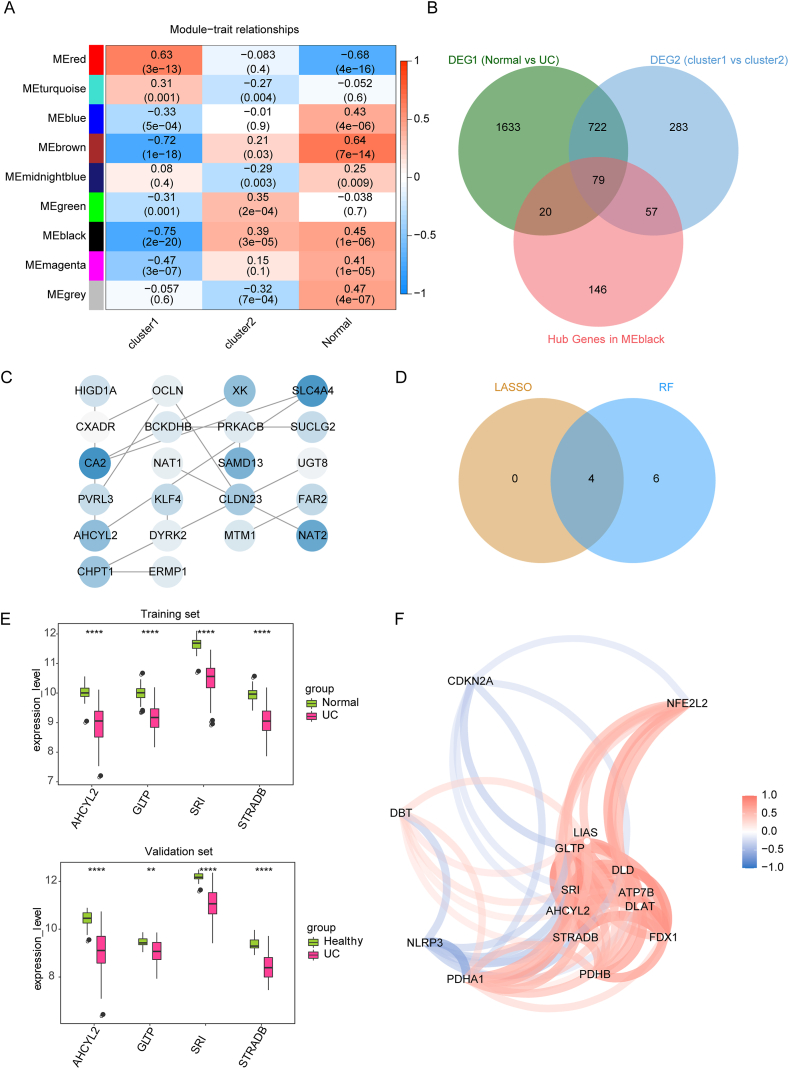
Network analysis of gene co-expression within distinct clusters and the normal group, alongside the selection of distinctive genes. (A) A heatmap visually representing module eigengenes and clinical traits association. (B) A Venn diagram illustrating the overlap of candidate genes among DEG1, DEG2, and hub genes. (C) An analysis of PPI networks, shedding light on key candidate genes. (D) A Venn diagram showcasing the genes shared between the LASSO regression and RF algorithm. (E) Boxplots showcasing four characteristic genes' expression pattern in UC and control samples within both the training and validation sets. (F) A Pearson correlation network illuminating the intricate relationships among the four characteristic genes and the eleven DE-CRGs.

WGCNA and differential expression analyses were performed to select the key candidate genes associated with different UC clusters and normal individuals. The black module that was most correlated with among cluster 1, cluster 2 and the normal group were selected (*P* < 0.05 and cor >0.35) (Fig. 5A), hence 302 genes under the cut-off criteria (|GS| > 0.2 and |MM| > 0.8) within the black module were considered as hub genes for further analysis (Fig. S1D). On the other hand, through analyzing differential gene expression in different clusters and by evaluating genes that differed in the UC and normal groups, a total of 2454 DEG1 in the UC and normal groups (Fig. S2A) and 1141 DEG2 in different clusters (Fig. S2B) were selected. Further, the intersection of DEG1, DEG2 and hub genes yielded 79 candidate genes (Fig. 5B). A PPI network (contained 22 nodes) for candidate genes was constructed to explore the protein linkages between them (Fig. 5C). Furthermore, using the LASSO algorithm (with ten-fold cross-validation and the optimal lambda = 0.04959348) (Figs. S3A–B) and the RF algorithm (Fig. S3C), four characteristic genes out of 79 candidate genes were screened out (Fig. 5D), namely, AHCYL2, GLTP, SRI, and STRADB. As shown in Fig. 5E, the expression of mRNAs encoded by four characteristic genes were found to be decreased in UC compared to normal samples from training and validation sets. Besides, the Pearson correlation network of four characteristic genes and eleven DE-CRGs suggested a most negatively association between AHCYL2 and NLRP3 expression, and a considerably positive correlation of STRADB and FDX1 (Fig. 5F–Table S2).

### 3.5 Diagnostic efficacy of the characteristic genes for UC

**Fig. 6:**
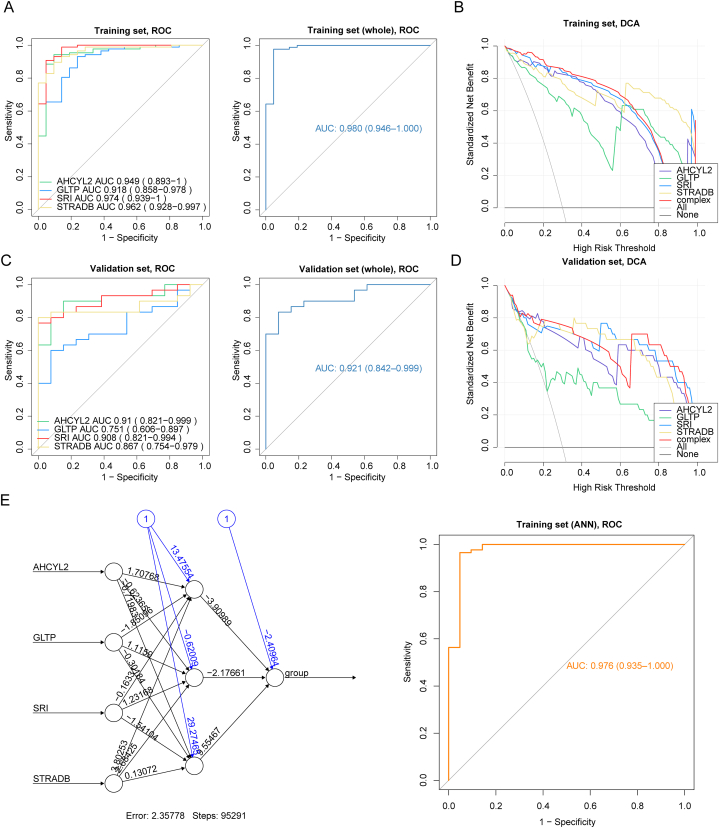
Diagnostic performance of the UC characteristic genes. (A) ROC analysis of characteristic genes as individuals and as a whole in the training set. (B) DCA curve of characteristic genes in the training set. (C) ROC analysis of characteristic genes as individuals and as a whole in the validation set. (D) DCA curve of characteristic genes in the validation set. (E) ANN model for validation of characteristic genes in the training set.

For diagnosis efficiency of the characteristic genes for UC, ROC curve analysis in the training set revealed that both four individuals and as a complex were effective in detecting UC (AUC >0.9) (Fig. 6A), as did in DCA plot (Fig. 6B). Evaluation of the diagnostic accuracy of these characteristic genes and as a whole in the validation set yielded AUCs above 0.75, suggesting that all four characteristic genes could predict UC well (Fig. 6C). In the DCA curve from the validation set, the predictive performance of characteristic genes was preferable (Fig. 6D). Further, the ANN model was built for validation of four characteristic genes, where the ROC analysis of ANN confirmed its excellent predictive efficacy for UC (Fig. 6E).

**Fig. 7:**
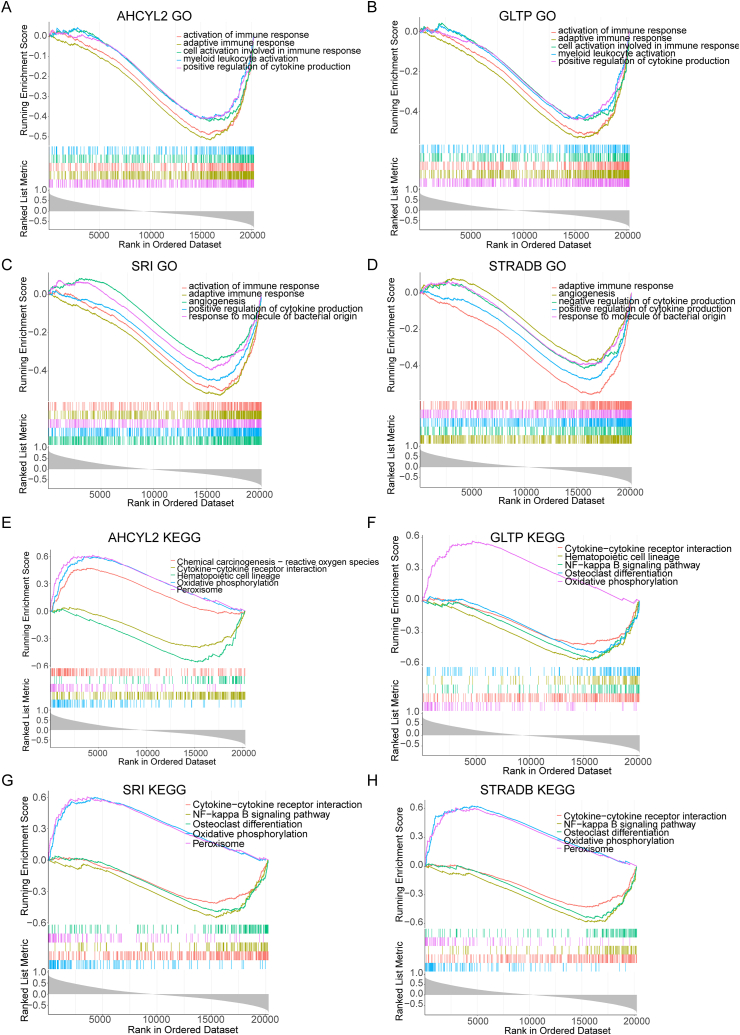
GSEA enrichment analysis of four genes diagnostic for UC. (A–D) Functional analysis of GO BP items of characteristic genes. (E–H) GSEA enrichment analysis in the KEGG pathway of characteristic genes.

Furthermore, the involvement of UC characteristic genes in potential signaling pathways was evaluated by GSEA. GO analysis showed that AHCYL2, GLTP, SRI, and STRADB were all negatively linked to immune responses (Fig. 7A–D). KEGG pathway showed that all of these UC characteristic genes were positively correlated with oxidative phosphorylation, and negatively associated with cytokine-cytokine receptor interaction (Fig. 7E–H).

### 3.6 Immune cell infiltration in UC

**Fig. 8:**
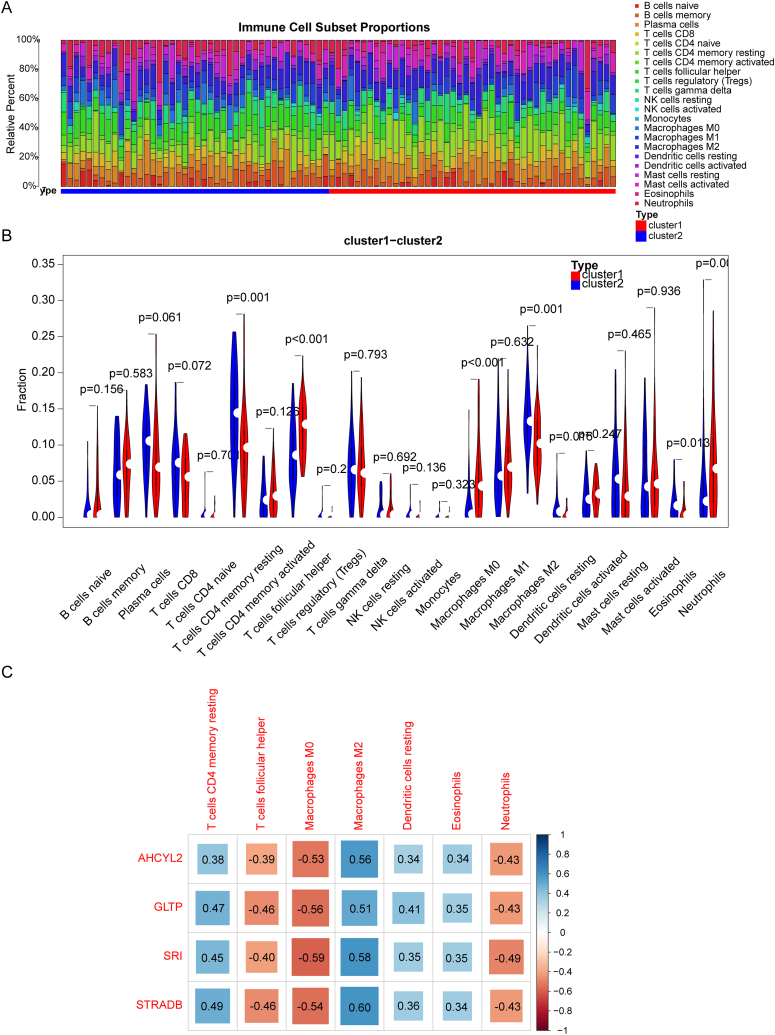
Analysis on immune microenvironments in the different clusters. (A) Heatmap showing the abundance of infiltrating immune cells in clusters 1 and 2. (B) Violin plot showing the differential infiltration of seven types of immune cells in the clusters. (C) Heatmap of the Spearman correlation between UC characteristic genes and differentially infiltrating immune cells, the size of the squares represents the Spearman's correlation coefficient.

A visual representation in the form of a heatmap showcased infiltration of 22 distinct immune cell types within each patient from the training set (Fig. 8A). Notably, the infiltration patterns of seven immune cell types showed notable disparities between cluster 1 and cluster 2. These included memory resting CD4 T cells, follicular helper T cells, M0 macrophages, M2 macrophages, resting dendritic cells, eosinophils, and neutrophils (Fig. 8B). In the exploration of the intricate relationships between characteristic genes and the variance in immune cell infiltration, it was observed that SRI expression revealed negative association with M0 macrophages infiltration. Conversely, STRADB demonstrated a notably positive association with M2 macrophages infiltration (Fig. 8C).

### 3.7 Expression of characteristic genes in UC patients and DSS-induced colitis

**Fig. 9:**
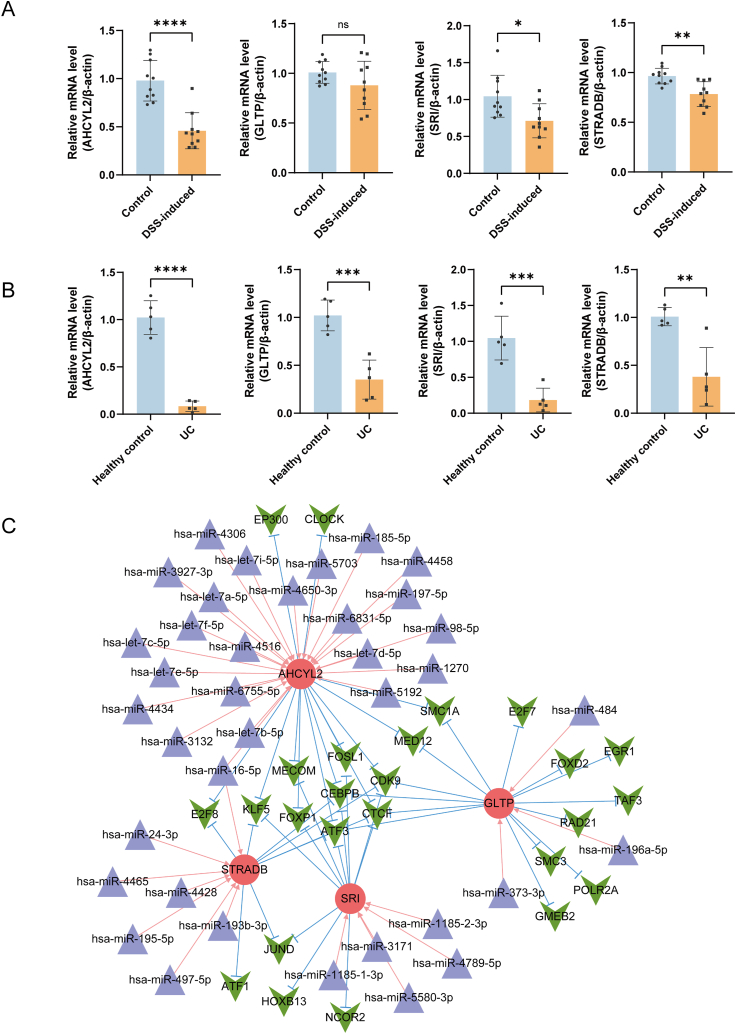
Expression of characteristic genes in UC patients and the miRNA-mRNA-TF network. (A) qRT-PCR analysis of the relative expression of the four characteristic genes in mice with DSS-induced and control mice. (B) Expression of the four characteristic genes in clinical samples. (C) The miRNA-mRNA-TF network of the four characteristic genes. Red circles represent mRNA, blue triangles represent miRNA, and green arrowheads represent TF. (For interpretation of the references to colour in this figure legend, the reader is referred to the Web version of this article.)

Further, the bioinformatics results were validated in clinical and mice samples by qRT-PCR. The levels of AHCYL2, SRI, and STRADB mRNAs were found to be lower in DSS-induced than in control mice, whereas the levels of GLTP mRNA did not differ in the two groups (Fig. 9A). In contrast, these genes were notably downregulated in UC samples than in normal colon tissue, aligning with public databases outcomes (Fig. 9B). To better understand inner mechanisms behind these characteristic genes in UC, miRNA-mRNA-TF network was constructed. This network was found to include 452 miRNA-mRNA-TF pairs, consisting of 37 miRNAs, 4 mRNAs, and 25 TFs (Fig. 9C).

## 4 Discussion

At present, due to the complex etiology of UC, conventional treatment strategies have limited effectiveness. Intestinal homeostatic imbalances are critical in the development of UC. The present study explored the potential association of cuproptosis with intestinal mucosal homeostasis of UC, determined molecular types in UC, and identified genes diagnostic of this condition. These findings extend our comprehension on cuproptosis in UC and provide insight into new-established therapeutic strategies.

Based on data from public databases, the present study found that the levels of six cuproptosis-promoting genes, namely PDHA1, DLD, PDHB, DLAT, FDX1, LIAS, were declined, whereas the expression of the cuproptosis-suppressing gene CDKN2A showed a rise, in UC patients than in healthy controls. These findings were consistent with previous results [12,13]. The levels of these cuproptosis-regulated proteins were validated in mice models of UC and clinical samples. The expression of FDX1, LIAS, and DLAT were found to be markedly reduced in the intestinal mucosa of DSS-induced than of control mice, suggesting that cuproptosis likely occurred in this animal model of UC. Similarly, evaluation of clinical samples demonstrated that LIAS, FDX1, and DLAT were notably changed in UC than in normal tissues. Moreover, these levels of expression may be associated with disease severity, as FDX1 expression level was increased in patients with mild UC than in controls, and LIAS and DLAT were inversely related to disease severity. As a pivotal regulator of cuproptosis, FDX1 could facilitate the occurrence of cuproptosis by reducing Cu^2+^ to the more cytotoxic Cu^1+^ and controlling protein lipoylation. Based on findings from both the mouse model and clinical samples, we postulated that cuproptosis levels might be heightened during the initial stage of the disease and diminish as the severity increases. However, the precise mechanism remains elusive and calls for more detailed investigation. In addition, complex mechanisms may underlie the regulation of DE-CRGs during UC development, resulting in differences between FDX1 mRNA and protein expression.

According to DE-CRGs expression profiles, UC patients could be divided into two clusters, with cluster 1 enriched for immune-related pathways and cluster 2 enriched for metabolism-related pathways. Specifically, cluster 1 showed significant increases in IL-17 production and dendritic cell (DC) migration. IL-17 cytokine family, which comprised IL-17A and IL-17F, participates in inflammatory responses by promoting pro-inflammatory cytokines and chemokines release, and IL-17D exhibits capability for maintaining intestinal homeostasis [[28], [29], [30]]. DCs are antigen presenting cells with different migration patterns that serve distinct immune functions [31]. Inhibition of plasmacytoid DC migration to isolated lymphoid follicles in the colon may improve DSS-induced colitis in mice [32]. Immune infiltration analysis of the two clusters within current research found that the infiltration of follicular T helper cells was notably elevated in cluster 1, whereas infiltration of M2 macrophages was higher in cluster 2. Follicular T helper cells can modulate B cell activation, with elevated numbers of follicular helper T cells enhancing the numbers of memory B cells and plasma cells, resulting in inflammatory factors release [33]. In contrast, M2 macrophages have anti-inflammatory roles in UC [34]. The results of the present study thus suggest that patients in cluster 1 exhibit higher levels of inflammation during UC, and that anti-IL17 A/F treatment may have therapeutic efficacy in these patients.

GSEA illustrated the strong enrichment of cluster 2 genes in ATP and membrane lipid metabolic processes. ATP metabolism, the primary source of cellular energy, was found to be significantly reduced in active UC [35], whereas increased synthesis of ATP was protective in mice with experimental colitis [36]. Membrane lipid homeostasis is important for maintaining intestinal barrier function. Imbalances of membrane lipids in mice could cause endoplasmic reticulum (ER) stress, promote the necroptosis of intestinal epithelial cells, and eventually lead to spontaneous colitis [37]. Therefore, nutritional supplementation may be more effective in cluster 2 patients, as their levels of metabolism are higher than in cluster 1 patients.

WGCNA and differential gene expression analysis obtained 79 candidate genes, with subsequent machine learning identifying four characteristic genes. ROC curve and DCA curves analysis found that all four characteristic genes were diagnostic for UC. The structure of AHCYL2, a member of the AHCY family, was highly similar to that of AHCYL1, although both AHCYL1 and AHCYL2 were unable to hydrolyze S-adenosyl-l-homocysteine (SAH) [[38], [39], [40]]. AHCYL1 was found to participate in the regulation of autophagy and ferroptosis [41,42], suggesting that AHCYL2 may perform similar functions. GLTP could mediate the intermembrane transfer of glycosphingolipids and play a role in UC by affecting the sphingolipid metabolism process [43]. SRI encodes a soluble resistance-related calcium-binding protein (sorcin), which was found to regulate calcium homeostasis and inhibit ER stress-induced apoptosis [44,45]. STRADB belongs to the STE20 family of serine/threonine kinases, knockout of STRADB was found to enhance CD8^+^ T cell infiltration in a tumor model of triple-negative breast cancer [46], and the interaction of STRADB with X chromosome-linked inhibitor of apoptosis protein (XIAP) could preserve cells from apoptosis [47]. Because cell death and immune infiltration play critical roles in the course of UC, determining the potential biological functions of these four characteristic genes in this chronic inflammatory disease merits further exploration.

Functional enrichment analysis illustrated, the all four characteristic genes were notably negatively related with adaptive immune responses and the positive regulation of cytokine production. Adaptive and innate immunity work together to maintain intestinal immune homeostasis, with the disruption of immune homeostasis exacerbating mucosal inflammation and barrier dysfunction, promoting the progression of UC [48,49]. Cytokines also serve as determinant in UC development, as imbalances between pro- and anti-inflammatory factors can lead to excessive immune responses in the intestinal mucosa, further compounding intestinal inflammation [50]. These results imply that the four characteristic genes may participate in UC pathogenesis by regulating immune responses. Immune cell infiltration analysis demonstrated, M0 macrophages were negatively associated with SRI, whereas M2 macrophages were positively correlated with STRADB. Intestinal macrophages are resident innate immune cells that are involved in the maintenance of barrier immunity [51]. To macrophages, both M1 and M2 are derived from M0, with M1 usually showing pro-inflammatory capabilities and M2 with anti-inflammatory capabilities, and being involved in tissue repair [52]. Therefore, STRADB may regulate infiltration of M2 macrophages, resulting in the suppression of mucosal inflammation or enhancement of intestinal repair. qRT-PCR analysis of samples from the mice model of UC and of clinical samples showed that the levels of AHCYL2, GLTP, SRI, and STRADB were marked decreased in UC than in normal tissue samples. These findings align with GEO database outcomes. Despite the limited reports on the relationship between the four characteristic genes and cuproptosis, our Pearson correlation network has identified a positive correlation between STRADB and FDX1. Conversely, the expression of AHCYL2 was found to negatively correlate with NLRP3 expression. Given these findings, we postulated that these characteristic genes could potentially regulate cuproptosis in UC.

The present study had several limitations. Most of the data were derived from public databases, limiting information on the included patients. One major limitation of this study is the relatively small sample size used for validation, which may introduce potential selection bias. Therefore, it is essential to validate our findings in larger sample sizes in future studies. Future studies should evaluate the levels of cuproptosis in different cell subsets, investigate the associations of different patient clusters with clinical features, and explore the specific mechanism of the four characteristic genes in the regulation of cuproptosis.

## 5 Conclusion

Current research found that CRGs were differentially expressed in UC patients and that these patients could be classified into two clusters according to DE-CRGs expression profiles. Four genes were identified as characteristic genes for the diagnosis of UC. These findings may contribute to unraveling the potential regulators of cuproptosis, identifying novel diagnostic genes for UC, and increasing the possibility of individualized precision treatment of UC.

## Funding

This work was supported by 10.13039/501100001809National Natural Science Foundation of China (81960108, 82170550, 82160107, 82260108); Applied Basic Research Projects of Yunnan Province (202201AY070001-048); Youth Talents Program of Yunnan Province (RLQB20220004); Medicine Leading Talent of Health and Family Planning Commission of Yunnan Province (L-201607); Yunnan Health Training Project of High Level Talents (H-2019050); 535 Talent Project of First Affiliated Hospital of 10.13039/501100003996Kunming Medical University (2023535D06, 2023533Q03).

## Ethics approval and consent to participate

All participants provided written informed consent and the study was approved by the Ethics Committee of The First Affiliated Hospital of Kunming Medical University (KYLL-2016011). Animal experiments in the study were approved by the Animal Ethics Committee of Kunming Medical University (KMMU2021187) and performed in compliance with the National Guidelines for the Care and Use of Animals.

## Data availability statement

The datasets presented in this study can be found in online repositories. The original contributions presented in the study are included in the article/supplementary material. Further inquiries can be directed to the corresponding author.

## CRediT authorship contribution statement

**Xinyu Bai:** Writing – original draft. **Fengrui Zhang:** Writing – review & editing. **Chan Zhou:** Methodology. **Jingxian Yan:** Methodology. **Hao Liang:** Validation. **Rui Zhu:** Validation. **Min Gong:** Validation. **Huixian Song:** Software. **Junkun Niu:** Conceptualization. **Yinglei Miao:** Conceptualization.

## Declaration of competing interest

The authors declare that they have no known competing financial interests or personal relationships that could have appeared to influence the work reported in this paper.
