# Supplementary material for: Identification of cuproptosis-related molecular classification and characteristic genes in ulcerative colitis
Source: Heliyon. 2024 Jan 19;10(2):e24875. doi: 10.1016/j.heliyon.2024.e24875 (PMC10835364; doi:10.1016/j.heliyon.2024.e24875)
Supplement: Multimedia component 1 [file mmc1.docx]

**Table S1**. Consensus clustering results of two cuproptosis-related clusters.

**Table S2**. Pearson correlation analysis of four diagnostic genes and eleven DE-CRGs.

**
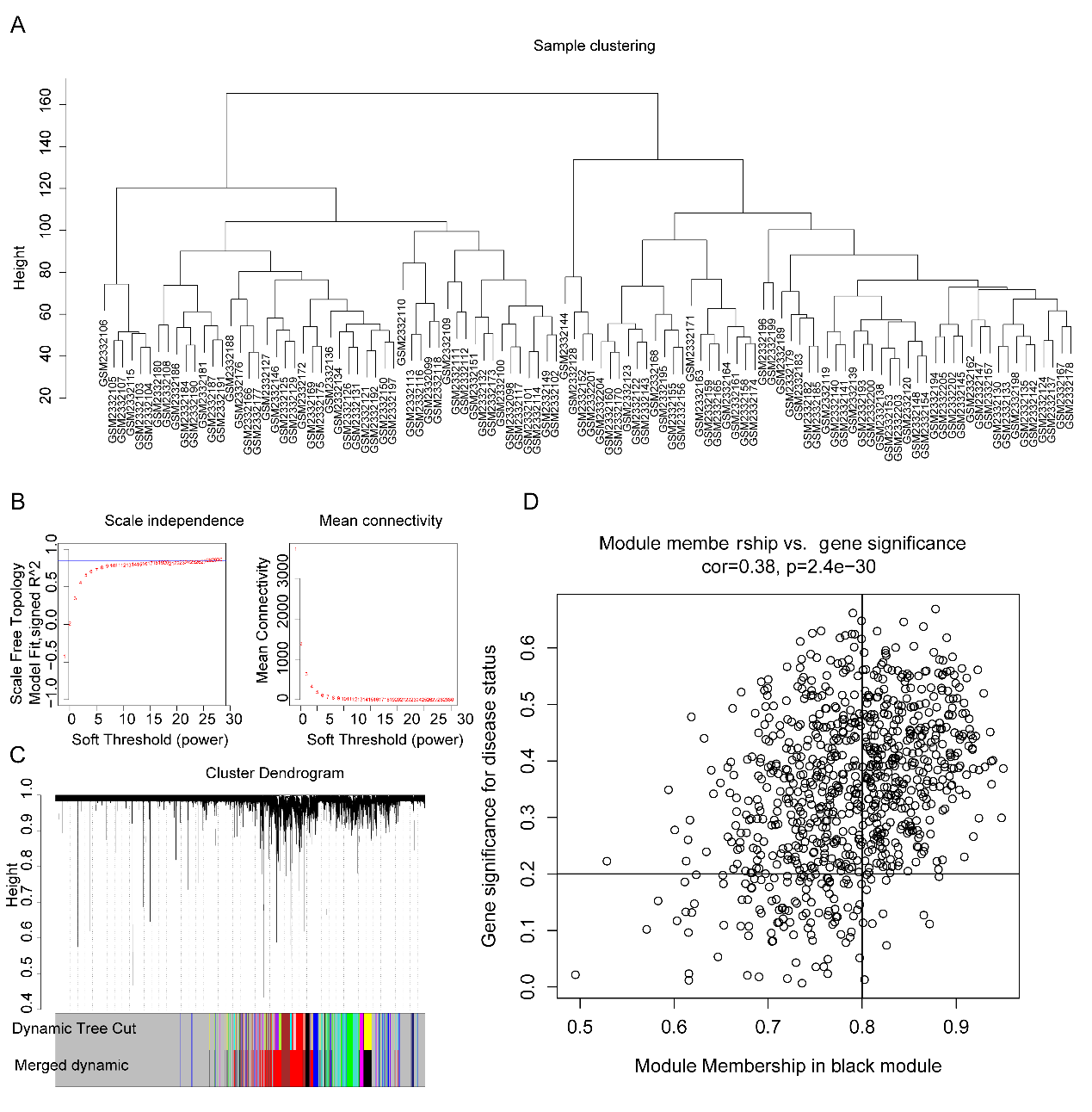
**

**Figure S1**. Weighted gene co-expression network analysis (WGCNA) and differential expression analysis in the training set. (A) Sample clustering analysis showing that there were no outlier samples. (B) Determination of soft-thresholding power. (C) Gene dendrogram showed nine co-expressed modules, each represented by a distinct color. (D) Scatterplot of module membership vs gene significance for differential UC clusters and the normal group in the black module.


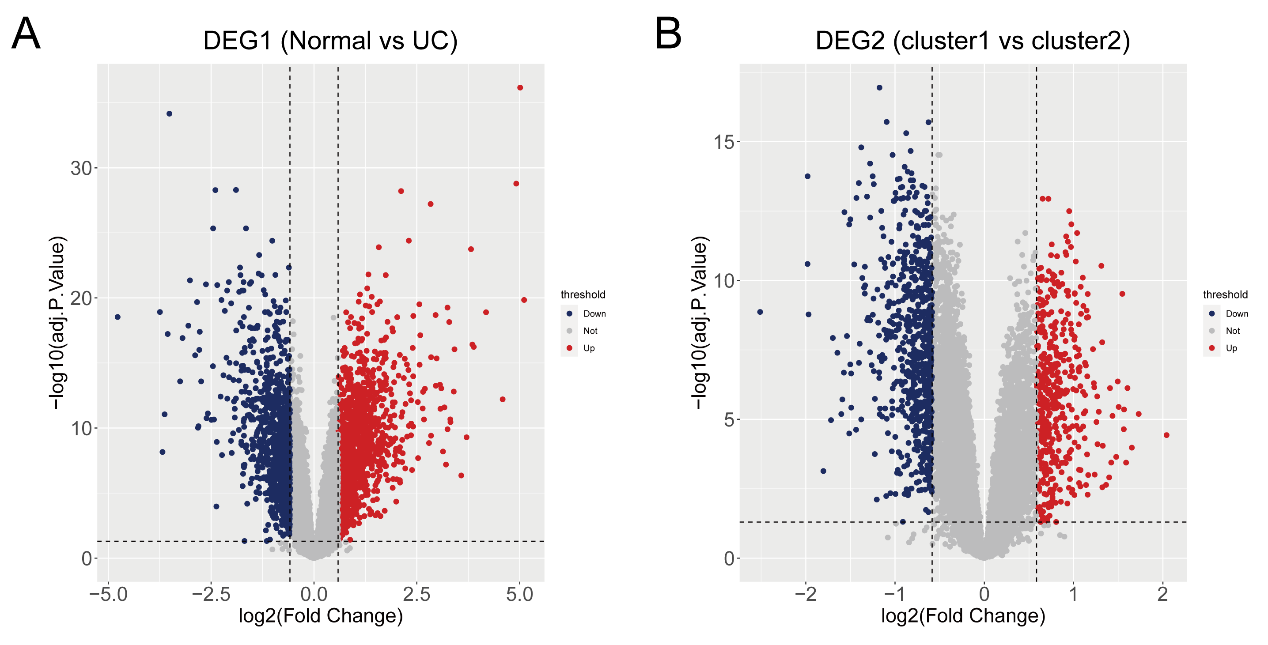


**Figure S2**. Differential expression analysis in different clusters and in the UC and normal groups. (A) Volcano plot showing DEG1 for UC patients and controls. (B) The volcano plot showed DEG2 for clusters 1 and 2.


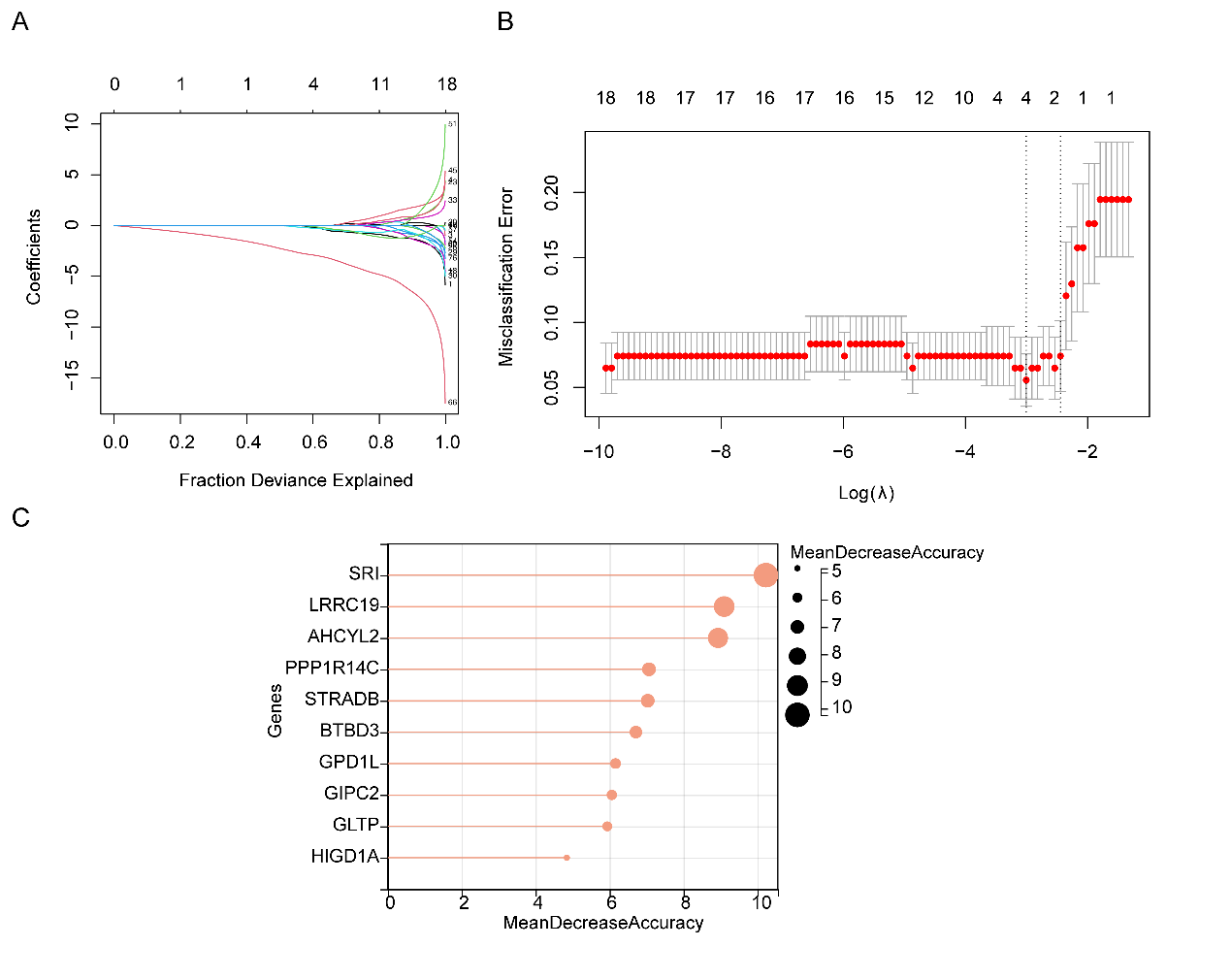


**Figure S3**. Machine-learning methods for screening diagnostic genes. (A, B) Determination of the penalization coefficient on LASSO regression analysis. (C) Lollipop plot showing the top 10 genes with different MeanDecreaseAccuracy in the RF model.
