# Supplementary material for: Identification of cuproptosis-related molecular classification and characteristic genes in ulcerative colitis
Source: Heliyon. 2024 Jan 19;10(2):e24875. doi: 10.1016/j.heliyon.2024.e24875 (PMC10835364; doi:10.1016/j.heliyon.2024.e24875)
Supplement: Multimedia component 2 [file mmc2.pdf]

Original images of western blotting

Because the detection of target protein and internal reference needs to be accomplished at the same time on the same membrane, the same membrane needs to be clipped. The corresponding markers for the target protein and internal reference shown are shown, both at the corresponding positions. Details are shown in the figure below. If possible, please esteemed editors to review the original bands and the manuscript we have provided so far.

The Marker used in this study was PageRuler Prestained Protein Ladder, 10 to 180 kDa (Thermo Fisher, THE-26616)

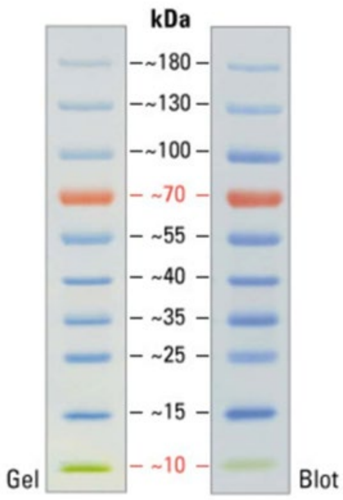

The original images of Figure 3D:

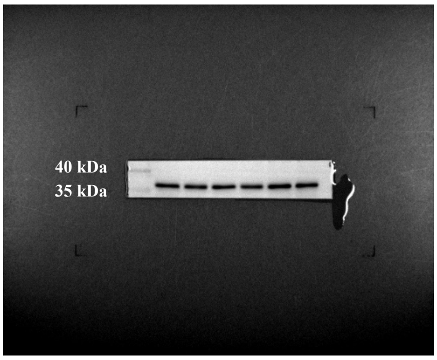

Protein name: GAPDH;

MW: 37kDa;

Left to right: control 1, control 2, control 3, DSS-induced 1, DSS-induced 2, DSS-induced 3

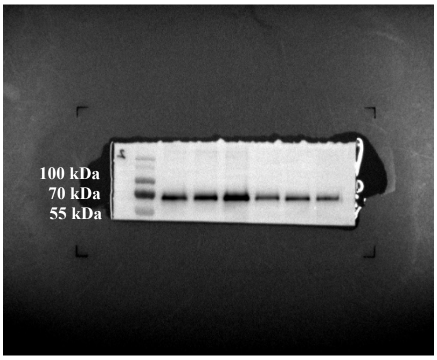

Protein name: DLAT;

MW: 70kDa;

Left to right: control 1, control 2, control 3, DSS-induced 1, DSS-induced 2, DSS-induced 3

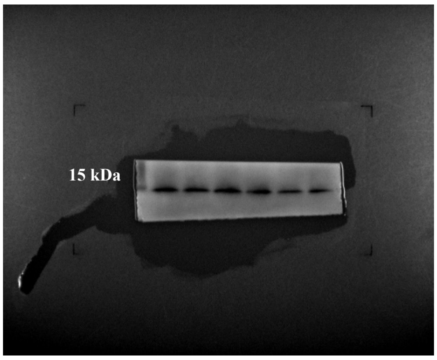

Protein name: FDX1;

MW: 13kDa;

Left to right: control 1, control 2, control 3, DSS-induced 1, DSS-induced 2, DSS-induced 3

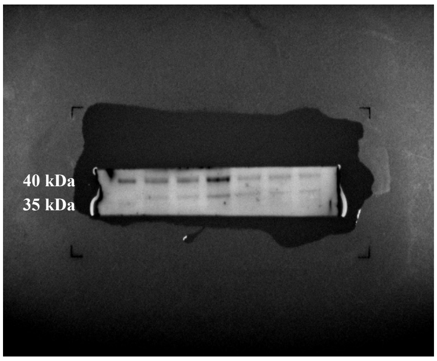

Protein name: LIAS;

MW: 40kDa;

Left to right: control 1, control 2, control 3, DSS-induced 1, DSS-induced 2, DSS-induced 3

The original images of Figure 3E:

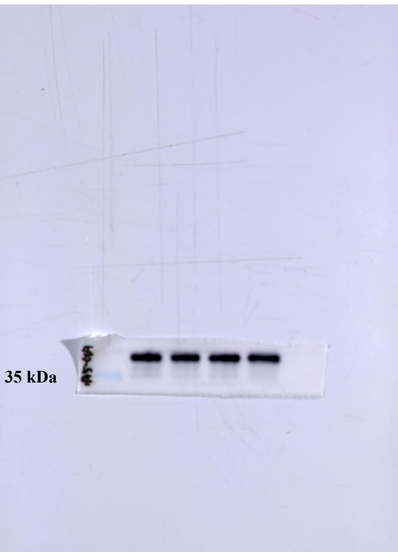

Protein name: GAPDH;

MW: 37kDa;

Left to right: Healthy control, Mild UC, Moderate UC, Severe UC

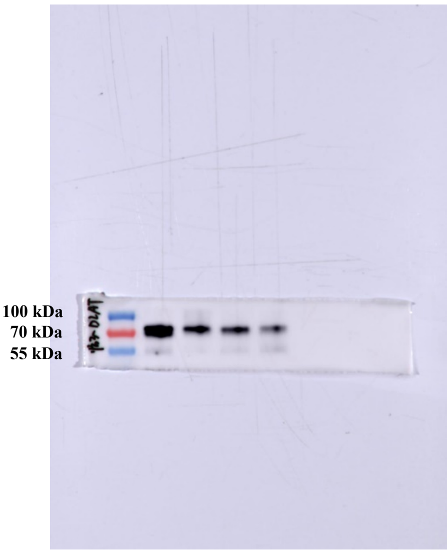

Protein name: DLAT;

MW: 70kDa;

Left to right: Healthy control, Mild UC, Moderate UC, Severe UC

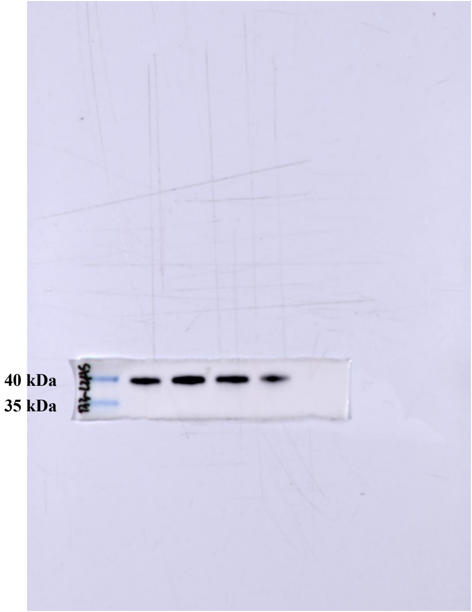

Protein name: LIAS;

MW: 40kDa;

Left to right: Healthy control, Mild UC, Moderate UC, Severe UC

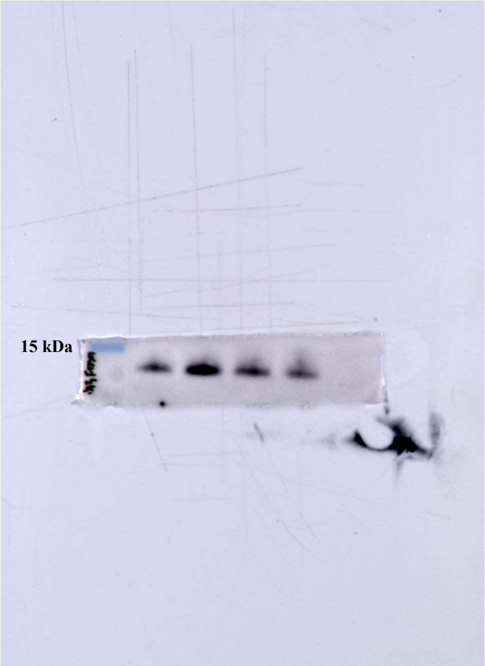

Protein name: FDX1;

MW: 13kDa;

Left to right: Healthy control, Mild UC, Moderate UC, Severe UC
